# Species Composition and Diversity of Malaria Vector Breeding Habitats in Trincomalee District of Sri Lanka

**DOI:** 10.1155/2015/823810

**Published:** 2015-10-25

**Authors:** Nayana Gunathilaka, Wimaladharma Abeyewickreme, Menaka Hapugoda, Rajitha Wickremasinghe

**Affiliations:** ^1^Molecular Medicine Unit, Faculty of Medicine, University of Kelaniya, P.O. Box 6, Thallagolla Road, Ragama, Sri Lanka; ^2^Tropical & Environmental Diseases & Health Associates, No. 3, Elibank Road, Colombo 5, Sri Lanka; ^3^Department of Public Health, Faculty of Medicine, University of Kelaniya, P.O. Box 6, Thallagolla Road, Ragama, Sri Lanka

## Abstract

*Introduction*. Mosquito larval ecology is important in determining larval densities and species assemblage. This in turn influences malaria transmission in an area. Therefore, understanding larval habitat ecology is important in designing malaria control programs. *Method*. Larval surveys were conducted in 20 localities under five sentinel sites (Padavisiripura, Gomarankadawala, Thoppur, Mollipothana, and Ichchallampaththu) in Trincomalee District, Eastern Province of Sri Lanka, between June 2010 and July 2013. The relationship between seven abiotic variables (temperature, pH, conductivity, Total Dissolved Solid (TDS), turbidity, Dissolved Oxygen (DO), and salinity) was measured. *Results*. A total of 21,347 anophelines were recorded representing 15 species. *Anopheles subpictus* 24.72% (5,278/21,347) was the predominant species, followed by 24.67% (5,267/21,347) of *An. nigerrimus* and 14.56% (3,109/21,347) of *An. peditaeniatus*. A total of 9,430 breeding habitats under twenty-one categories were identified. *An. culcicifacies* was noted to be highest from built wells (20.5%) with high salinity (1102.3 ± 81.8 mg/L), followed by waste water collections (20.2%) having low DO levels (2.85 ± 0.03 mg/L) and high TDS (1,654 ± 140 mg/L). *Conclusion*. This study opens an avenue to explore new breeding habitats of malaria vectors in the country and reemphasizes the requirement of conducting entomological surveillance to detect potential transmission of malaria in Sri Lanka under the current malaria elimination programme.

## 1. Background

Mosquitoes are insects of the order Diptera and family Culicidae [1]. They are responsible for the spread of a wide range of diseases including malaria, yellow fever, dengue, West Nile virus, and Rift Valley fever [2–4]. These mosquito-borne diseases, infecting more than 700 million people around the world each year, result in as many as two million deaths annually [5].

One of these diseases, malaria, is transmitted between humans by adult female mosquitoes of the genus* Anopheles*. Malaria is endemic in tropical and subtropical regions where it causes over 300 million acute illnesses and at least one million deaths each year [6]. Malaria has been a scourge in Sri Lanka from ancient times [7]. Recently, Sri Lanka has achieved a significant reduction in malaria incidence since 2000 and embarked on a malaria elimination phase in 2009 of a substantial progress toward elimination of malaria in the last decade [8].

During the years of 2011 and 2012 there were only 124 and 23, respectively, of indigenous malaria cases. Remarkably, the numbers of* Plasmodium falciparum* cases during these years were limited to five (in 2011) and four (in 2012). Furthermore, the majority (*n* = 99) reported in 2011 were personnel from the security forces who were engaged in post-civil war rehabilitation and reconstruction work in the northern and eastern parts of the country, which indicated the presence of pockets of active transmission at that time. Another notable feature during the last few years is the steady increase in the proportion/numbers of imported malaria cases with India and Africa being the common source countries. This trend continues to date with no indigenous malaria cases reported during the year 2013.

At present, Sri Lanka is the only country in South Asia with such ambitious goals that, in fact, the country has almost accomplished according to the national malaria surveillance data [9]. The objectives of the elimination drive include elimination of indigenous* P. falciparum* malaria by year 2012, elimination of indigenous* Plasmodium vivax* malaria by 2014, maintenance of a zero mortality of malaria cases, and prevention of reintroduction of malaria into the country [9, 10].

Several strategies have been implemented such as early diagnosis and prompt treatment of malaria patients and asymptomatic parasite carriers, implementation of selective and sustainable vector control measures based on the principles of integrated vector management protocols, forecasting, early detection, prevention of outbreaks and the rapid and effective containment of outbreaks, regular reassessment of the country's malaria situation, enhancement of community participation and partnership building for effective and sustainable malaria control, promotion of human resource development and capacity building, and promotion of operational research in order to achieve the stated objectives [10].

However, improving the entomological surveillance was considered a major challenge under the malaria elimination program, especially in the northern and eastern provinces of the country that experienced a terror war that lasted for almost three decades.

Mosquitoes exploit almost all types of lentic aquatic habitats for breeding. The breeding habitat is crucial for mosquito dynamics, because it is the location where many important life cycle processes take place: oviposition, larval development, emergence and probably resting, swarming, and mating [11].

Observations on local anopheline mosquitoes and their breeding habitats were first studied in 1927 [12]. It was maintained that the chief breeding places of* Anopheles* were paddy fields, where larvae were found throughout the year in varying densities. The commonest species present therein were* Anopheles subpictus*,* Anopheles barbirostris*,* Anopheles nigerrimus*,* Anopheles peditaeniatus,* and* Anopheles culicifacies* which were more abundant in running water containing aquatic vegetation. The breeding of* An. culicifacies* and* An. subpictus* was extensively found in wells, abandoned paddy fields, pools, and burrow pits [12].

There was not any published study thereafter in these early malaria endemic areas on species habitat diversity and species composition due to many reasons. The separatist war which raged for last 30 years in the north and east of the country was one of them. Since the end of the separatist war, Sri Lanka has been on a steep development trajectory with the building of new industries and hotels and resettling people in areas that were previously endemic for malaria; the construction of roads traversing the country; increasing global business investments; and a rapidly growing tourist industry, all of which are associated with increased travel of foreign nationals, resettlements, expanding agricultural activities, and introduction of foreign labour into the country. Ongoing construction projects are leading to the creation of new vector breeding sites, including previously endemic areas.

The principal vector of malaria,* An. culicifacies*, and secondary vectors such as* An. subpictus*, are as prevalent in the country as previously [13]. Furthermore,* An. culicifacies* has reportedly diversified its breeding habitats from previously clear, unpolluted slow flowing waters to more polluted and still sources of water [14].

Therefore, mosquito larval habitat ecology is important in determining larval densities, relative importance of breeding habitats, and species assemblage as well as designing mosquito control programs [15].

Hence, the main objectives of this study were to determine the larval composition of anopheline mosquitoes in different breeding habitats categories and to detect the water quality status of each breeding habitat. The present study also focused on assessing changes in the anopheline mosquito fauna and replacement of mosquito larval habitats in Trincomalee District which was formerly a malaria endemic area in the Eastern Province of Sri Lanka.

The results obtained from the present study may be essential in updating the knowledge about mosquito breeding habitats and designing of efficient strategies for mosquito control under the current malaria elimination phase in Sri Lanka.

## 2. Methods

### 2.1. Study Area

**Figure 1:**
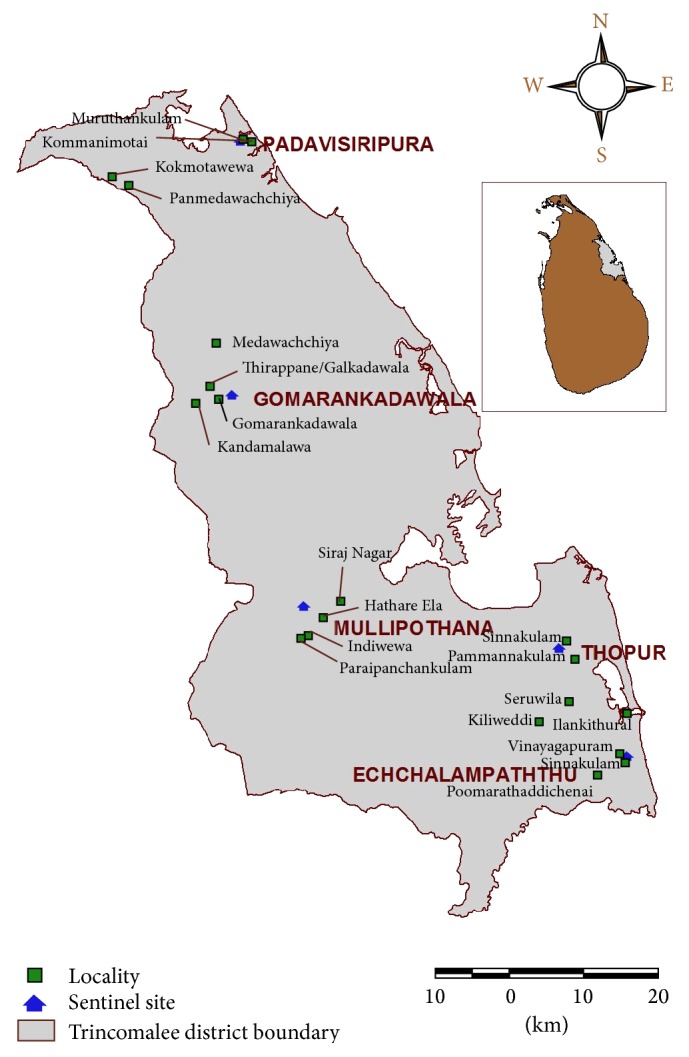
Map showing sentinel sites and localities in the District of Trincomalee.

Malaria was formerly an endemic problem in the District of Trincomalee, Eastern Province of Sri Lanka. The entomological survey was carried out for 3 consecutive years (June 2010 to June 2013) from different localities in the District of Trincomalee. A total of 20 study areas under five entomological sentinel sites, namely, Padavisiripura, Gomarankadawala, Thoppur, Mollipothana, and Ichchallampaththu, within a radius of about 20 km were selected (Figure 1).

Factors such as past malaria history, environmental conditions, availability of breeding sites, an established agricultural community, and feasibility of field operations to collect relevant data were considered in selecting study areas.

### 2.2. Description of the Breeding Habitats

Breeding sits were categorized and classified where water movement was expressed as running, moderate, and stagnant water. Vegetation was observed on each sampling whereas duck-weed, water hyacinths, algae, emerging plants, standing plants, and grasses were identified. All potential breeding habitats were identified in all 20 localities through a preliminary survey conducted for a period of one month prior to the research study and certain fixed and temporary breeding places were identified for the larval survey.

### 2.3. Mosquito Sampling

Collection of immature mosquitoes was made by dipping methods as per World Health Organization (WHO) guidelines [16]. A minimum of 50 dips were taken from each breeding habitat depending on the size of the breeding place using standard dippers (250 mL capacity). Large plastic pipettes and small white enamel pans were used for small and shallow water bodies. The* Anopheles* larvae were separated from the Culicine larvae and classified as early instar stage (I and II) or late instar stage (III and IV). The pupae were also collected.

### 2.4. Sample Identification

Collected stages III and IV larvae were placed individually in a depression microscopic slide with a minimum amount of water and identified under a light microscope with an objective (×10) using standard morphological keys developed for Sri Lankan anopheline larvae [17]. Stages I and II instar larvae were reared to reach III and IV instar larvae and identified in species level [18, 19].

Collected pupae were temporary mounted on microscopic slide and view at magnification up to 100x under a light microscope (Olympus Optical Co. Ltd., Tokyo). Mounted pupae were identified using a standard key developed for* Anopheles* pupae [20]. Much emphasis was given to the characters such as pupal trumpet, puddle, and more prominent abdominal seta.

### 2.5. Collection of Water Samples

Three water samples were collected into glass collecting bottles separately from each breeding habitat between 09:00–12:00 hr on each sampling day concurrently with the larval surveys.

### 2.6. Analysis of Water Quality Parameters

Seven abiotic variables, temperature, hydrogen ion concentration (pH), conductivity, Total Dissolved Solids (TDS), turbidity, salinity, and Dissolved Oxygen (DO), were measured on-site at the time of collection. Temperature (portable meter, Hach SenSION TM), pH (portable meter, Hach SenSION TM), and DO (digital meter EUTECH Dowp 300/02K) and conductivity, TDS, and salinity were also measured (Hach SenSION TM multi probe meter).

### 2.7. Data Analysis

The density of mosquito larvae in each breeding habitat was calculated using the following formula [21]:

(1) $$\begin{array}{c} D=\frac{l}{L}\cdot 100\%, \end{array}$$

where *D* is density, *l* is number of specimens of each mosquito species, and *L* is number of all specimens.

The following density classes were accepted [21]:

  Satellite species (*D* < 1%).
  Subdominant species (1 < *D* < 5%).
  Dominant species (*D* > 5%).

The species richness, Shannon-Wiener diversity index (Equation (2)), and Pielou's evenness index (Equations (3) and (4)) were determined separately for the detected mosquito larval species at the study sites:

(2) $$\begin{array}{c} H^{\prime }=-\overset{R}{\underset{i=1}{\sum }}p_{i}\ln p_{i}. \end{array}$$

*H*′ is the Shannon-Wiener diversity index and *p*_*i*_ is the proportion of characters belonging to the *i*th type of letter in the string of interest. Consider

(3) $$\begin{array}{c} J^{\prime }=\frac{H^{\prime }}{H_{\max}^{\prime }}. \end{array}$$

*J*′ is Pielou's evenness index, where *H*′ is the number derived from Shannon-Wiener diversity index and *H*_max⁡_′ is the maximum value of *H*′, which is given by

(4) $$\begin{array}{c} H_{\max}^{\prime }=-\overset{S}{\underset{i=1}{\sum }}\frac{1}{S}\ln\frac{1}{S}=\ln S. \end{array}$$

Physicochemical properties in different breeding habitats were examined by Kruskall-Wallis Analysis of Variance (ANOVA) followed by Turkey's multiple comparison test [22] using MINITAB 14.0 software package. Relationships between abundance and physicochemical variables in breeding habitats were examined by correlation analysis. Values with *P* < 0.05 were considered as statistical significant correlations.

## 3. Results

A total of 21,347 anophelines were recorded representing 15 species from 598,046 dips in 2,721 breeding places (Table 1). Morphological identification revealed 24.72% (*n* = 5,278) belonging to* Anopheles subpictus* Complex, followed by 24.67% (*n* = 5,267)* Anopheles nigerrimus*, 14.56% (*n* = 3,109)* Anopheles peditaeniatus*, 9.25% (*n* = 1,975)* Anopheles barbirostris*, 6.69% (*n* = 1,430)* Anopheles pallidus,* 5.0% (*n* = 1,068)* Anopheles culicifacies*, 4.86% (*n* = 1,038)* Anopheles annularis*, 4.59% (*n* = 980)* Anopheles vagus*, 3.02% (*n* = 645)* Anopheles varuna*, 1.3% (*n* = 278)* Anopheles barbumbrosus*, 0.38% (*n* = 81)* Anopheles pseudojamesi*, 0.37% (*n* = 79)* Anopheles jamesii*, 0.25% (*n* = 53)* Anopheles aconitus*, 0.24% (*n* = 52)* Anopheles tessellatus,* and 0.07% (*n* = 14)* Anopheles maculatus*.

Mosquito larvae were found in 21 types of water collections. The relative abundance of each mosquito species encountered in different breeding habitats is presented in Table 1. In this study, animal footprints, rain water pools, tyre marks, burrow pits, quarry pits, and rock pools were observed as temporary breeding sites with discolored and stagnant water that usually dry up during hot months. These sites were found in open areas, streets, yards, and other sites in villages and towns.

*Marshy lands* are deep lowland and water-logged areas adjacent to ponds or irrigation lands. These breeding places are usually shaded and characterized by natural vegetation and highly organic materials. Marshy lands were recorded as the eighth most abundant breeding habitat for anophelines (1,283 mosquito larvae were found).* An. nigerrimus* was the commonest species found in this site, where the relative abundance was 32.5% (417/1,283), followed by 27.2% (349/1,283) of* An. peditaeniatus* and 17.8% (229/1,283) of* An. barbirostris. An. culicifacies* represented only 0.2% (2/1,283) of the total collection.

*Main canals* are vertical irrigational canals distributed in agricultural lands. These sites were storm drains, which receive water to agricultural fields mainly in paddy fields. These habitats were positive for 12 anopheline species in which 47.5% (533/1,121) comprised* An. subpictus*, followed by 14.8% (166/1,121) of* An. nigerrimus. An. culicifacies* accounted for 1.8% (20/1,121) of all anophelines.

*Field canals* are artificial site that receives irrigation water from the main canals.* An. nigerrimus *was the most common species in this site, where the relative abundance was 29.3% (163/556), followed by* An. peditaeniatus* 24.8% (138/556). Only 1.6% (9/556) of the total collection comprised* An. culicifacies*.

*Ponds* were widely distributed in the study areas. These sites were depressed grounds filled with water from flooded irrigated lands or damaged canal banks. Pools are semipermanent breeding site with stagnant water.* An. subpictus* was the most common species at these sites, whereas the relative abundance was 44.1% (642/1,457), followed by that of* An. nigerrimus* (15.6% (228/1,457)).* An. culicifacies* comprised only 1.0% (15/1,457) of the total collection.

*Canals* are the structures used for conveyance of water for irrigation. Canals with vegetation were permanent breeding sites, water flow was moderate and chocking vegetation (water hyacinths) with other organic litter was standing on the sides of canals with grasses. Only 2.0% (1/49) of* An. culicifacies* was recorded from this breeding habitat category. The most abundant species was* An. barbirostris* (38.8% (19/49)), followed by 14.3% (7/49) each of* An. nigerrimus* and* An. subpictus*.

*Paddy fields* were seasonal breeding places, widely distributed in agricultural lands. Rice fields were surveyed for mosquito larvae during different phases of plant growth. The water was clear and stagnant. This habitat was noted as the third most abundant breeding habitat for anophelines.* An. subpictus* (28.3% (1,036/3,655)) was the most common species found in this type of breeding sites, followed by 25.4% (927/3,655)* An. nigerrimus* and 18.3% (669/3,655) of* An. peditaeniatus*. The relative abundance of* An. culicifacies* was 0.1% (2/3,655).

*Drains containing waste water* were the fifth largest breeding habitat observed in the Trincomalee District, where a total of 2,226 of anopheline larvae collected. This type of breeding site was usually neglected having foul-smelling water filled with vegetation (water hyacinths, duckweed, and algae) and other matters such as debris, polythene, and empty cans.* An. subpictus* was the most common species at this site, whereas the relative abundance was 53.8% (1,197/2,226), followed by 20.2% (450/2,226) of* An. culicifacies.* This breeding site was the second most abundant site for* An. culicifacies*.

*Wells* were detected as the most abundant and second most abundant habitat. In general, four types of wells were observed such as earth wells, built wells, common wells, and agricultural wells. Cement bounded wells, used only for drinking, bathing, or domestic purposes, were considered as built wells. Earth wells used only for drinking, bathing, or domestic purposes were regarded as unbounded wells. Bounded or unbounded wells used only for agricultural purposes were considered as agricultural wells. Further, bounded or unbounded wells used by the community were considered as common wells.

Wells are permanent breeding sites and usually contained clean and clear water except agricultural wells in which mud, leaves, algae, debris, and garbage were observed sometimes. Breeding of anopheline larvae in built wells (*n* = 4,484) was only second to breeding in tank margins (*n* = 6,687). Wells were the most conducive breeding habitat of* An. culicifacies* (20.5% (920/4,484)) in the Trincomalee District.

*Tank margins* were the most abundant mosquito breeding habitat noted in the District of Trincomalee. Of the 15 anopheline species, only breeding of* An. maculatus* was not recorded from this breeding habitat. The majority of 30.0% (2,005/6,687) was represented by* An. nigerrimus*. Only 0.1% (8/6,687) of* An. culicifacies* was represented from the District of Trincomalee.

Anopheline breeding was observed in* water storage tanks*, which were barrel shaped or overhead tanks to store drinking water and generally made of plastic. The relative abundance of* An. culicifacies* was 7.7% in the District of Trincomalee, the majority being* An. peditaeniatus* (39.7% (31/79)).

The calculated species richness (*S*), Shannon-Wiener diversity index (*H*′), and Pielou's evenness index (*J*′) at the study sites in the District of Trincomalee are included in Table 1. As suggested by the results, the lowest degree of species evenness (categorized by Pielou's evenness index-*J*′) was indicated by common well (*J*′ = 0.02), while the highest degree of species evenness was indicated by the breeding habitats at the canals with vegetation (*J*′ = 0.87). The domestic built wells (*H*′ = 1.96) indicated the highest species diversity (categorized by Shannon-Wiener diversity index-*H*′ = 0.02) while the common wells indicated the lowest degree of species diversity (Table 1). The rain water pools (*S* = 15) tend to indicate the highest species richness (*S*) while the common wells (*S* = 1) indicated the lowest.

In total, 9,430 breeding habitats were analyzed for seven physicochemical parameters. The mean physicochemical characteristics of water in breeding habitat types along with the Kruskall-Wallis Analysis of Variance (ANOVA) followed by Turkey's multiple comparison tests are given in Table 2. In general,* An. barbirostris* and* An. peditaeniatus* were recorded from all types of breeding habitats.* An. peditaeniatus* and* An. nigerrimus* species were found positive more in breeding habitats associated with agricultural ecosystems. There is a correlation between anopheline densities of each species and physicochemical characteristics of water in the breeding habitats. Physicochemical properties in each type of breeding habitat were significantly correlated with each other.

The DO levels in rock pools, earth wells, common wells, and agricultural wells were significantly different. The DO levels of waste water collections were low compared to other breeding habitat categories (2.85 ± 0.03 mg/L). Tyre marks and animal foot prints were also noted with similar characteristics. There was a significant difference in the pH of rock pools, earth wells, agricultural wells, animal foot prints, and quarry pits. The highest mean TDS level was detected from waste water collections (1,654 ± 140 mg/L) ranging from 274 to 4,721 milligram per liter (mg/L).

## 4. Discussion

Descriptive entomological studies have not been done in most parts of the country for a long time. The only study which covered entomological aspects of malaria vectors in North and Eastern Provinces of Sri Lanka including the District of Trincomalee was the study conducted by Carter in 1927 [12].

Comparison of the anopheline mosquito population compositions of two surveys in both past (1924–1927) and present (2010–2013) clearly shows the changes taking place in mosquito species in the District of Trincomalee. The present study confirms that the mosquito replacement after 86 years of time is due to environmental changes caused by urbanization, resettlements, and development projects in the form of increased mosquito breeding habitats.

*Anopheles culicifacies* was encountered in a variety of breeding habitats in addition to the traditional breeding habitats noted in the country by previous researches [23–25]. The built wells and waste water collections encountered the majority of* An. culicifacies* throughout the study period. Waste water collections were found in semiurbanized areas, which contained stagnant water in blocked drains.* Anopheles subpictus*,* Anopheles barbirostris*,* Anopheles peditaeniatus*,* Anopheles nigerrimus*, and* Culex tritaeniorhynchus* (Giles) were bred along with* An. culicifacies* in these habitats. Water bodies with similar characteristics were found in all localities in the district and all these habitats were highly positive for* An. subpictus*.

The DO level of the waste water in drains was below 3 mg/L. According to this value, it can be categorized as third class (polluted) surface water based on the standards available for surface water [26]. Some studies conducted in Sri Lanka, as well as in India and Pakistan, have shown the abundance of* An. culicifacies* to be positively associated with DO and more prominent in water bodies with high DO [27]. Therefore, this indicates that anophelines including* An. culicifacies* can tolerate breeding habitats with low DO levels.

The built wells, which were positive for* An. culicifacies,* were mostly used for drinking and bathing purposes. There were no* An. culicifacies* larvae found in abandoned built wells and agricultural wells. Some studies conducted in the Trincomalee District of Sri Lanka have shown* Anopheles* breeding in wells, which were not used [12], but this study provided an opposite behavior of anopheline mosquitoes in selecting wells as the breeding habitats for their oviposition.

The possible explanation as to why* An. culicifacies* larvae were frequently found in domestic water containing drains and wells may be due to two reasons: firstly, the preferential section of open habitats for oviposition by anophelines [28] and, secondly, the reason that larval predation may be less prevalent in temporary and man-made habitats than it is in large, permanent habitats [29].

The importance of wells and waste water drains as breeding places for* Anopheles* indicates that both of these habitats act as larval reservoirs during dry seasons. Of the wells present, approximately 90% of them were cement bounded; they vary considerably in depth; but many do not dry out during the dry season. In addition, most of the built wells that were positive for anophelines were being used. Therefore, this creates a serious threat on malaria transmissions because most of these wells were located at close proximity to human habitations. Hence, breeding habitats such as wells and waste water containing drains can be acted as important breeding places even in the dry seasons.

The salinity levels in breeding habitats were high and exceeded the threshold of 200–300 mg/L [30]. This study observed that the salinity levels were very high in ground water habitats, especially, in built wells (1102.3 ± 81.8 mg/L) and earth wells (1227 ± 196 mg/L). Since this water is used for drinking purpose, it may have some health impact on humans in these areas. This is also a good indication that the* An. culicifacies* can breed in water containing high salinity since the majority of* An. culicifacies* was recorded from built wells.

Some studies have shown that an increasing access to water supply pipes in individual houses, the construction of ponds for fisheries, vegetable farming, road access in villages to vegetable marketing, a frequent movement of vehicles, and microhydropower electricity gridlines in communities might have created more conducive environments for the passive and active dispersal of mosquitoes in the highlands of Nepal [31, 32]. The irrigation canal system and irrigation practices may influence the breeding of anopheline vectors by creating stagnant water pools within the irrigation system's boundaries, when water is not flowing through the system [33, 34].

The current study also found main canals, field canals, canals with vegetation, marshy lands, and paddy fields as anopheline breeding habitats associated with rice cultivations and irrigational ecosystems.* Anopheles culicifacies* was noted from all the habitat categories mentioned above.

In paddy fields, the relative prevalence of the larvae of different species varied somewhat according to the condition of the fields. The most evident variations were associated with* An. nigerrimus*,* An. peditaeniatus,* and* An. subpictus*. The mosquito larvae were relatively most numerous when the crop was well grown and least numerous during the period of cultivation and early growth of the crop when the water surface was wholly or partially exposed.* An. subpictus*, on the other hand, was not abundant in the stagnant and usually turbid water present during the intervals between ploughing and until the crop was thoroughly established when it decreased considerably in numbers. Three of these species were prevalent in fallow fields containing water, and the results obtained for each were more or less intermediate between the extremes found for the different categories.

Ability of the major vector and other potential vectors to occur in a variety of habitats may hinder the current vector controlling programs. Unusual breeding habitats such as waste water collections and brackish water habitats may mislead the larval controlling activities. As a result of that the ecological disturbance, which is a direct result of human activity, may also increase the number of breeding sites.

The current study observed 15 anopheline species. Vector incrimination studies done for most of these anopheline species were reported to play a role in malaria transmission [35]. Therefore, the prevalence of major and potential vectors with diversified breeding habitats implies a continuing high receptivity and vulnerability to malaria in previously endemic areas. This, when combined with the increasing reports of imported malaria from diverse parts of the country, almost certainly points to a sustained high risk of malaria reintroduction unless rigorous measures are taken to prevent it.

Hence, further attention should be drawn towards controlling the larval breeding especially on anthropogenic and natural breeding habitats in an environmentally friendly manner. To facilitate the planning of environmentally friendly control measures, there is a need for further investigations on breeding habitat diversity and ecology of anopheline mosquito developmental stages. The precise nature of the necessary antilarval works will vary in different places and will be largely dependent upon the results of the entomological investigations made. Therefore, it is warranted to device appropriate vector controlling measure by the Ministry of Health through proper entomological investigations.

## 5. Conclusions

There is a significant change in the breeding habitats which may have resulted due to human activities due to unplanned development projects together with creation of unattended stagnant water bodies. This study opens an avenue to explore new breeding habitats of malaria vectors in the country and reemphasizes the requirement of conducting entomological surveillance to detect potential transmission of malaria in Sri Lanka under the current malaria elimination programme.

## Figures and Tables

**Table 1:** Abundance of *Anopheles* mosquito species in habitats measured for physicochemical variables in the District of Trincomalee.

Breeding habitats	Diversity indices			Species density														
	*H*′	*S*	*J*′	*An. culicifacies*	*An. subpictus*	*An. peditaeniatus*	*An. pallidus*	*An. vagus*	*An. varuna*	*An. barbirostris*	*An. annularis*	*An. aconitus*	*An. jamesii*	*An. nigerrimus*	*An. barbumbrosus*	*An. tessellatus*	*An. pseudojamesi*	*An. maculatus*
Tank margin	1.98	14	0.75	0.1	14.2	19.4	8.1	4.9	3.0	8.7	9.1	0.6	0.6	30.0	0.8	0.03	0.48	0
Lake margin	1.76	11	0.73	0.0	32.6	16.9	3.1	4.1	0.1	16.5	2.6	0.0	0.2	20.7	3.1	0.16	0	0
Canal with vegetation	1.69	7	0.87	2.0	14.3	12.2	0.0	6.1	12.2	38.8	0.0	0.0	0.0	14.3	0.0	0	0	0
Marshy land	1.72	11	0.72	0.2	8.9	27.2	3.8	5.5	1.7	17.8	0.7	0.2	1.6	32.5	0.0	0	0	0
Field canal	1.82	10	0.79	1.6	12.8	24.8	6.8	0.2	9.4	12.4	2.3	0.4	0.0	29.3	0.0	0	0	0
Main canal	1.68	12	0.68	1.8	47.5	11.7	2.3	6.9	2.8	9.5	1.9	0.4	0.4	14.8	0.0	0	0.09	0
Paddy field	1.87	12	0.75	0.1	28.3	18.3	4.1	6.1	0.2	7.1	6.3	0.0	2.2	25.4	1.3	0	0.58	0
Pond	1.71	12	0.69	1.0	44.1	13.0	7.1	2.9	0.8	11.1	1.6	0.0	0.3	15.6	2.3	0.07	0	0
Rock pool	1.73	10	0.75	1.3	40.1	10.7	7.8	15.7	3.6	2.8	1.0	0.0	0.2	16.8	0.0	0	0	0
Earth well (domestic)	1.68	12	0.67	1.7	45.1	11.5	2.5	3.7	0.1	12.1	3.2	0.0	0.2	17.3	2.5	0.07	0	0
Built well (domestic)	1.96	12	0.79	20.5	30.3	10.0	1.6	3.0	4.8	12.5	1.9	0.1	0.8	11.9	2.6	0	0	0
Common well	0.02	1	0.02	0.0	100.0	0.0	0.0	0.0	0.0	0.0	0.0	0.0	0.0	0.0	0.0	0	0	0
Agricultural well	0.11	2	0.16	0.0	97.6	0.0	0.0	0.0	0.0	2.4	0.0	0.0	0.0	0.0	0.0	0	0	0
Burrow pit	1.86	14	0.71	0.7	25.5	18.2	2.8	6.3	1.1	15.3	1.4	0.1	0.7	25.0	2.8	0.17	0	0.04
Animal footprint	1.31	7	0.67	0.0	54.2	5.7	0.9	11.9	0.0	5.7	0.0	0.0	0.0	21.1	0.4	0	0	0
Rain water pool	1.86	15	0.69	1.1	33.9	21.4	2.0	4.9	4.1	13.8	2.5	0.2	0.2	13.5	0.0	0.16	2.19	0.08
Quarry pit	1.89	11	0.79	3.9	0.8	11.5	22.5	1.0	2.9	15.1	0.8	0.3	22.7	18.5	0.0	0	0	0
River margin	1.33	7	0.68	0.0	56.5	22.0	3.8	3.8	0.0	6.2	4.3	0.0	0.0	3.3	0.0	0	0	0
Tyre mark	0.65	5	0.40	0.0	4.0	2.0	4.0	84.0	0.0	6.0	0.0	0.0	0.0	0.0	0.0	0	0	0
Waste water collection	1.33	8	0.64	20.2	53.8	3.0	15.3	1.3	0.0	0.5	1.0	0.0	0.0	5.0	0.0	0	0	0
Water storage tank	1.47	7	0.75	7.7	1.3	39.7	0.0	0.0	0.0	12.8	3.8	0.0	2.6	32.1	0.0	0	0	0

*H*′: Shannon-Wiener diversity index.

*S*: species richness.

*J*′: Pielou's evenness.

**Table 2:** Physicochemical characteristics (mean ± SE, range) of breeding habitats in the District of Trincomalee.

Breeding places	Temperature (°C)	DO (mg/L)	pH (25°C)	Conductivity (*µ*s/cm)	Salinity (mg/L)	TDS (mg/L)	Turbidity (NTU)
Tank margin	31.23 ± 0.08^a^	5.65 ± 0.07^a^	7.57 ± 0.03^a^	751.9 ± 65.4^a,c^	393.2 ± 37.5^a^	533.2 ± 46.1^a^	84.8 ± 14.0^a^
	(29.2–34.2)	(3.18–8.56)	(6.37–8.74)	(90.9–2874)	(15.2–1973)	(26–1987)	(0.47–1332.8)

Lake margin	31.64 ± 0.07^a^	5.73 ± 0.03^a^	7.74 ± 0.04^a^	748 ± 79.65^a,c^	352 ± 26.87^a^	539 ± 57.40^a^	95.9 ± 19.76^a^
	(29.5–33.6)	(3.73–6.32)	(6.95–8.53)	(85.92–1863)	(46.8–984)	(64.97–1538)	(7.84–763.8)

Canal with vegetation	31.42 ± 0.52^a^	4.97 ± 0.75^d^	7.64 ± 0.05^c^	352.1 ± 10.52^a^	138.2 ± 7.42^a^	257.1 ± 7.542^a^	27.92 ± 2.86^a^
	(29.5–33.62)	(3.57–5.58)	(6.78–7.85)	(318–529)	(97.86–263)	(198.9–328)	(7.92–89.63)

Marshy land	30.72 ± 0.13^a,c^	4.89 ± 0.13^d^	7.52 ± 0.04^b,c^	838.2 ± 33.6^a^	397.5 ± 14.7^a^	546.2 ± 25.5^a^	30.06 ± 1.83^a^
	(30.1–31.3)	(4.12–5.82)	(7.2–7.81)	(543–990)	(322–478)	(432–672)	(12.5–38.6)

Field canal	31.47 ± 0.15^a^	5.04 ± 0.11^d^	7.49 ± 0.06^c^	443.4 ± 21^a^	186 ± 10.1^a^	286 ± 13.1^a^	85.7 ± 27.7^a^
	(29.3–33.2)	(3.47–5.92)	(6.55–8.01)	(235–768)	(19.3–356)	(178.2–471)	(12.3–1110.3)

Main canal	31.26 ± 0.15^a^	4.98 ± 0.09^d^	7.44 ± 0.05^c^	392.9 ± 11.7^a^	170.8 ± 6.13^a^	272.02 ± 6.36^a^	25.25 ± 1.85^a^
	(29.4–33.7)	(3.48–5.72)	(6.59–7.93)	(320–584)	(123–258)	(212–354)	(9.38–67.2)

Paddy field	31.9 ± 0.11^e^	5.04 ± 0.0^d^	7.40 ± 0.05^c^	411.4 ± 14.4^a^	160.42 ± 5.73^a^	263.82 ± 7.71^a^	90.4 ± 16.0^a^
	(31.0–34.5)	(4.07–6.33)	(6.45–7.94)	(236–691)	(14.9–362)	(26–463)	(12.2–466.0)

Pond	31.55 ± 0.21^a,e^	5.01 ± 0.1^d^	7.43 ± 0.05^c^	555.43 ± 8.15^a^	205.26 ± 9.58^a^	337.77 ± 3.93^a^	48.22 ± 3.58^a^
	(30.1–33.9)	(4.19–5.96)	(6.86–7.94)	(438–673)	(132.8–298)	(303–388)	(6.2–78.4)

Rock pool	31.1 ± 0.41^a,d^	4.71 ± 0.1^b,d^	7.53 ± 0.08^b,c^	358.92 ± 5.59^a^	166.2 ± 7.92^a^	253.8 ± 6.23^a^	18.2 ± 1.36^a^
	(29.4–32.6)	(4.27–5.38)	(7.25–7.93)	(328–393)	(124.8–196.3)	(233–284)	(11.6–25.7)

Earth well	30.01 ± 0.21^c^	4.93 ± 0.13^a,d^	7.47 ± 0.06^a,c^	3655 ± 728^c^	1227 ± 196^c^	2197 ± 412^c^	6.57 ± 1.17^b^
	(28.4–31.0)	(4.17–5.52)	(7.15–7.86)	(503–8764)	(239–2154)	(381–5244)	(0.37–12.48)

Built well	30.54 ± 0.077^b^	4.10 ± 0.06^b^	7.52 ± 0.03^b^	2358 ± 174^b^	1102.3 ± 81.8^b,d^	1580 ± 115^b^	2.78 ± 0.41^b^
	(28.9–32.4)	(2.35–5.72)	(6.28–8.45)	(320–8530)	(133–3487)	(210–4632)	(0.01–21.4)

Common well	31.13 ± 0.12^a,b,c^	3.82 ± 0.1^b,c^	7.78 ± 0.11^b^	1626.3 ± 77.4^a,b,c^	819.7 ± 42.2^a,b,c^	1099.1 ± 64.7^a,b,c^	0.55 ± 0.06^a,b^
	(30.4–31.6)	(3.45–4.52)	(7.24–8.35)	(1312–1964)	(636–972)	(845–1398)	(0.19–0.85)

Agricultural well	30.03 ± 0.22^b,d,c^	4.77 ± 0.25^a,c,d^	7.62 ± 0.1^a,b,c^	1239 ± 149^a^	485.8 ± 45^a^	699 ± 54.6^a^	11.05 ± 3.95^a^
	(29.3–30.7)	(3.58–5.39)	(7.33–8.05)	(845–1875)	(322–673)	(537–863)	(1.12–27.3)

Burrow pit	31.16 ± 0.168^a^	5.865 ± 0.355^a^	7.47 ± 0.05^a^	810 ± 123^a,c^	398.1 ± 63.9^a^	565.2 ± 85.6^a^	131.3 ± 19.2^a^
	(29.4–32.5)	(3.34–9.3)	(6.62–8.33)	(236–1894)	(98.4–980)	(134.3–1364)	(5.3–326)

Animal foot print	31.73 ± 0.73^f^	2.53 ± 0.01^e^	7.48 ± 0.03^e^	590 ± 43.87^a^	316 ± 38.65^a^	425 ± 48.83^a^	65.49 ± 97.63^b^
	(29.74–34.52)	(0.46–3.72)	(7.14–7.92)	(287–864)	(123–673)	(243–718)	(13.85–982)

Rain water pool	31.25 ± 0.61^a^	4.21 ± 0.02^b^	7.63 ± 0.03^e^	528 ± 12.83^a^	318 ± 13.81^a^	443 ± 10.23^a^	19.83 ± 2.06^a^
	(30.3–33.6)	(3.91–5.17)	(7.08–7.92)	(321–872)	(198.23–523)	(218–672)	(9.83–28.75)

Quarry pit	31.88 ± 0.13^a,e^	4.57 ± 0.10^b^	7.61 ± 0.04^b,c^	361.95 ± 7.21^a^	166.46 ± 6.29^a^	266 ± 9.9^a^	29.62 ± 1.13^a^
	(30.7–32.6)	(3.65–5.92)	(7.3–7.92)	(318–435)	(123.8–245)	(211–371)	(22.4–38.75)

River margin	31.70 ± 0.02^a,e^	3.56 ± 0.16^b,c^	7.48 ± 0.01^e^	749 ± 24.70^a^	350 ± 8.92^a^	472 ± 10.51^a^	13.72 ± 7.62^a^
	(30.8–33.2)	(3.02–4.73)	(6.91–7.83)	(482–851)	(219–521)	(328–731)	(6.91–22.85)

Tyre mark	31.95 ± 0.04^f^	2.18 ± 0.03^e^	7.35 ± 0.05^e^	529 ± 75.32^a^	98.54 ± 24.3^a^	254 ± 132.9^a^	83.74 ± 9.43^b^
	(29.63–34.52)	(0.95–2.96)	(7.17–7.82)	(319–952)	(53.86–254.8)	(168.66–538.8)	(18.94–741.0)

Waste water collection	31.62 ± 0.78^f^	2.85 ± 0.03^e^	7.72 ± 0.08^e^	753.0 ± 53.1^a^	328 ± 24.97^a^	1654 ± 140^b^	37.1 ± 8.63^b^
	(29.64–34.21)	(0.84–3.83)	(7.08–8.93)	(397–1397)	(164.9–976)	(274–4721)	(2.85–386)

Water storage tank	30.8 ± 0.06^b^	4.77 ± 0.04^b^	7.57 ± 0.001^b^	2649 ± 152^b^	1325 ± 67.92^b,d^	527 ± 152.8^a^	2.92 ± 0.02^b^
	(29.81–33.91)	(3.87–5.94)	(7.01–7.93)	(361–6219)	(153–3417)	(263.3–1238)	(0.02–12.83)

Note: values are given to the nearest significant decimal. Different superscript letters in a row show significant differences (*P* < 0.05) indicated by Tukey's multiple comparison after Kruskall-Wallis test.

## References

[1] Service M. W. (2004). *Medical Entomology for Students*.

[2] Shaalan E. A.-S., Canyon D. V. (2009). Aquatic insect predators and mosquito control. *Tropical Biomedicine*.

[3] Maguire M., Skelly C., Weinstein P., Moloney J. (1999). Simulation modelling of *Aedes aegypti* prevalence, an environmental hazard surveillance tool for the control of dengue epidemics. *International Journal of Environmental Health Research*.

[4] Hay S. I., Cox J., Rogers D. J. (2002). Climate change and the resurgence of malaria in the east African highlands. *Nature*.

[5] Fradin M. S. (1998). Mosquitoes and mosquito repellants: a clinician's guide. *Annals of Internal Medicine*.

[6] World Health Organization (WHO) (2004). *The Global Burden of Disease: 2004 Update*.

[7] Samarasinghe L., Ramasamy R. (1990). A situation analysis of malaria in Sri Lanka. *Current Status of Malaria Research in Sri Lanka*.

[8] World Health Organization (WHO) (2011). The use of anti-malarial drugs. Report of a WHO informal consultation.

[9] http://www.malariacampaign.gov.lk/Downloads/final%20Annual%20Report%202011.pdf.

[10] Karunaweera N. D., Galappaththy G. N., Wirth D. F. (2014). On the road to eliminate malaria in Sri Lanka: lessons from history, challenges, gaps in knowledge and research needs. *Malaria Journal*.

[11] Overgaard H. J., Tsuda Y., Suwonkerd W., Takagi M. (2002). Characteristics of *Anopheles minimus* (Diptera: Culicidae) larval habitats in northern Thailand. *Environmental Entomology*.

[12] Carter H. F. (1927). Report on malaria and anopheline mosquitoes in Ceylon. *Ceylon Sessional Papers*.

[13] Premaratne R., Ortega L., Janakan N., Mendis K. N. (2014). Malaria elimination in Sri Lanka: what it would take to reach the goal. *South-East Asian Journal of Public Health*.

[14] Gunathilaka N., Fernando T., Hapugoda M., Wickremasinghe R., Wijeyerathne P., Abeyewickreme W. (2013). *Anopheles culicifacies* breeding in polluted water bodies in Trincomalee District of Sri Lanka. *Malaria Journal*.

[15] Simsek F. M. (2004). Seasonal larval and adult population dynamics and breeding habitat diversity of *Culex theileri* Theobald, 1903 (Diptera: Culicidae) in the Glba. district, Ankara, Turkey. *Turkish Journal of Zoology*.

[16] World Health Organization (WHO) (1992). *Entomological Field Techniques for Malaria Control Part 1 Learner's Guide*.

[17] Gunathilaka N., Fernando T., Hapugoda M., Abeyewickreme W., Wickremasinghe R. (2014). Revised morphological identification key to the larval anopheline (Diptera: Culicidae) of Sri Lanka. *Asian Pacific Journal of Tropical Biomedicine*.

[18] Amerasinghe F. P. (1990). A guide to the identification of anopheline mosquitoes (Diptera; Culicidae) of Sri Lanka.1 adult females. *The Ceylon Journal of Science (Biological Sciences)*.

[19] Padhn G. (2014). *Distribution of major and potential malaria vectors in Mannr and Trincomalee districts and systematics of anophelines in Sri Lanka (Ph.D Thesis [Ph.D. thesis]*.

[20] Amerasinghe F. (2013). A guide to the identification of the anopheline mosquitoes of Sri Lanka.111. Pupae. *Journal of the National Science Foundation of Sri Lanka*.

[21] Banaszak J., Winiewski H. (1999). Podstawyekologii. *Foundation of Ecology*.

[22] Zar J. H. (1984). *Bio Statistical Analysis*.

[23] Amerasinghe F. P., Ariyasena T. G. (1990). Larval survey of surface water-breeding mosquitoes during irrigation development in the Mahaweli Project, Sri Lanka. *Journal of Medical Entomology*.

[24] Amerasinghe F. P., Konradsen F., Fonseka K. T., Amerasinghe P. H. (1997). Anopheline (Diptera: Culicidae) breeding in traditional tank based village ecosystem in north central Sri Lanka. *Journal of Medical Entomology*.

[25] Kusumawathie P. H. D., Wickremasinghe A. R., Karunaweera N. D., Wijeyaratne M. J. S., Yapabandara A. M. G. M. (2006). Anopheline breeding in river bed pools below major dams in Sri Lanka. *Acta Tropica*.

[26] Rydzanicz K., Lone E. (2003). Species composition and seasonal dynamics of mosquito larvae in the Wrocław, Poland area. *Journal of Vector Ecology*.

[27] Amarasinghe F. P., Indrajith N. G., Ariyasena T. G. (1995). Physico-chemical characteristics of mosquito breeding habitats in an irrigation development area in Sri Lanka. *Ceylon Journal of Science (Biological Sciences)*.

[28] Bentley M. D., Day J. F. (1989). Chemical ecology and behavioral aspects of mosquito oviposition. *Annual Review of Entomology*.

[29] Sunahara T., Ishizaka K., Mogi M. (2002). Habitat size: a factor determining the opportunity for encounters between mosquito larvae and aquatic predators. *Journal of Vector Ecology*.

[30] World Health Organization (WHO) (1984). *Guidelines for Drinking—Water Quality; Volume 2. Health Criteria and Other Supporting Information*.

[31] Shrestha S. L. (1985). Dynamics of malaria transmission with reference to development projects in Nepal. *The Journal of Communicable Diseases*.

[32] Dhimal M., Ahrens B., Kuch U. (2014). Species composition, seasonal occurrence, habitat preference and altitudinal distribution of malaria and other disease vectors in eastern Nepal. *Parasites & Vectors*.

[33] Sharma V. P., Srivastava A., Nagpal B. N. (1994). A study of the relationship of rice cultivation and annual parasite incidence of malaria in India. *Social Science and Medicine*.

[34] Premasiri D. A. R., Wickremasinghe A. R., Premasiri D. S., Karunaweera N. (2005). Malarial vectors in an irrigated rice cultivation area in southern Sri Lanka. *Transactions of the Royal Society of Tropical Medicine and Hygiene*.

[35] Perera M. D. B., Hemingway J., Karunaratne S. H. P. P. (2008). Multiple insecticide resistance mechanisms involving metabolic changes and insensitive target sites selected in anopheline vectors of malaria in Sri Lanka. *Malaria Journal*.

